# A case of late-onset bleeding from an intercostal artery pseudoaneurysm after hemostasis using soft coagulation

**DOI:** 10.1186/s40792-024-01851-8

**Published:** 2024-02-28

**Authors:** Rie Shimizu, Kenichi Suda, Toshiki Takemoto, Shota Fukuda, Masato Chiba, Masaki Shimoji, Junichi Soh, Tetsuya Mitsudomi, Yasuhiro Tsutani

**Affiliations:** Division of Thoracic Surgery, Department of Surgery, Kindai University Faculty of Medicine, 377-2 Ohno-Higashi, Osaka-Sayama, 589-8581 Japan

**Keywords:** Postoperative complications, Postoperative bleeding, Hemoptysis, Soft coagulation, Angiography

## Abstract

**Background:**

The use of soft coagulation is becoming common in thoracic surgery. Soft coagulation provides rapid hemostasis from small vessels during surgery by dehydrating tissue and denaturing proteins, without burning the tissue.

**Case presentation:**

A 68-year-old man, with a history of right lower lobectomy 3 years prior, underwent a partial resection of the right upper lobe for a pulmonary nodule suspicious for secondary lung cancer. During the surgery, dissection of the adhesion caused a bleeding from the 6th intercostal artery, and hemostasis was achieved using soft coagulation (some degree of tissue carbonization was noticed at later mortality and morbidity conference). He experienced hemoptysis at postoperative day 18 and was transferred to our hospital. Contrast-enhanced CT scan revealed bleeding from the pseudoaneurysm of the 6th intercostal artery. Embolization was performed by angiography to stop the bleeding.

**Conclusions:**

We experienced a case of late-onset bleeding from a pseudoaneurysm related to soft coagulation hemostasis. Lessons learned from this patient are that additional hemostasis, such as ligation, would be considered for small arteries after hemostasis has been achieved by soft coagulation, especially when some degree of tissue carbonization is suspected.

## Background

Lung cancer surgery in Japan is becoming safer as a result of multiple factors, including the development of surgical instruments, improved perioperative management, and the increase of small/peripheral lung nodules [1–3]. However, serious complications may still occur after lung cancer surgery, including postoperative hemorrhage, bronchopleural fistula and pyothorax, and brain strokes, although the frequencies of these events are quite low. Postoperative hemorrhages are usually observed during the early postoperative period, and delayed hemorrhage may occur in some rare cases. The proportion of postoperative hemorrhage cases that require reoperation is approximately 0.6–1.3% [4–6]. Here we report a case of a lung cancer patient with late-onset postoperative hemorrhage (postoperative 18th day) after partial resection of the lung.

## Case presentation

**Fig. 1:**
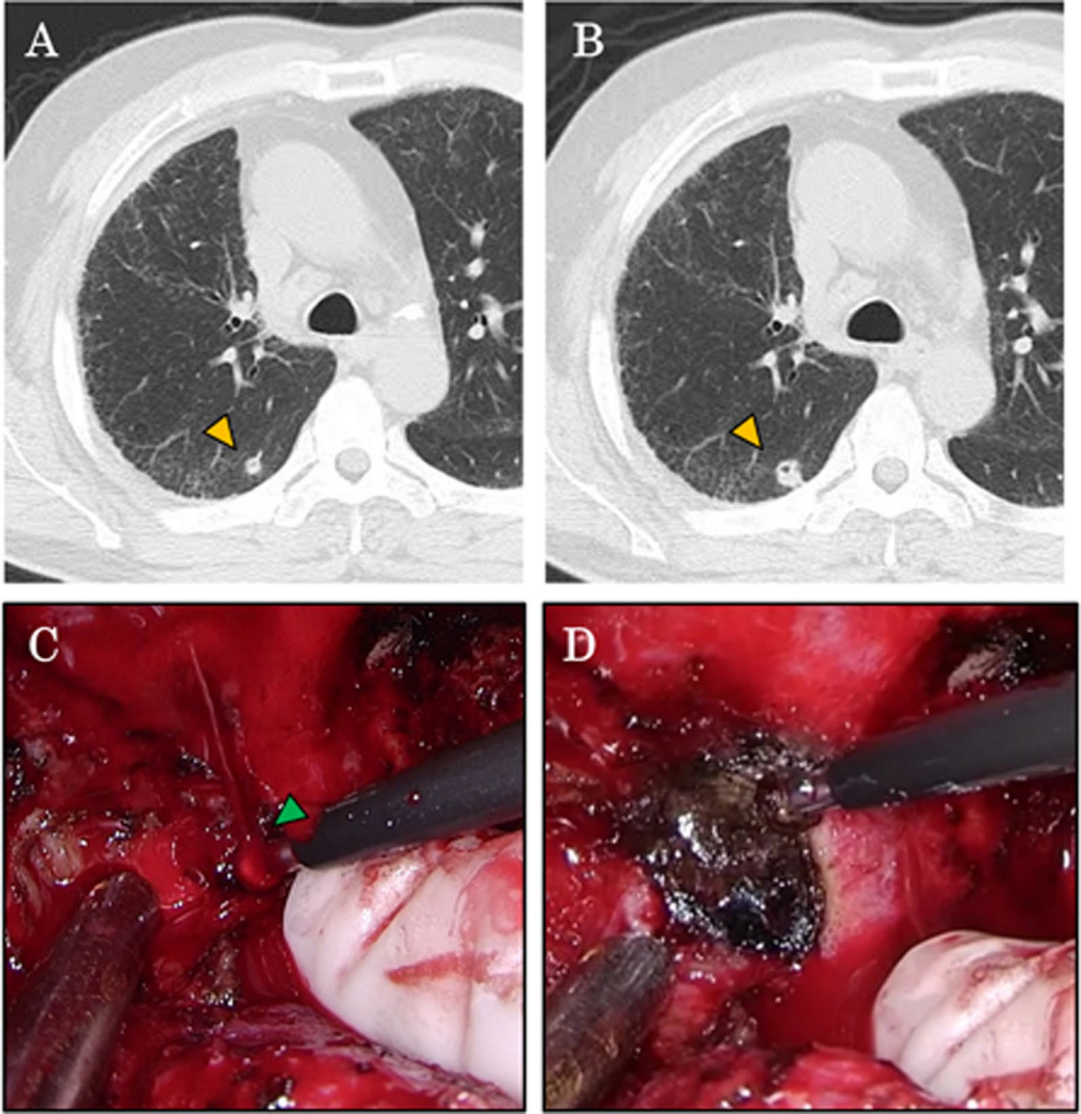
The enlarged pulmonary tumor and intraoperative bleeding and hemostasis. A second primary lung cancer identified during postoperative follow-up after right lower lobectomy (December 2018) (**a**) that enlarged slightly 6 months later (June 2019) (**b**). Partial resection was planned for the pulmonary nodule. Intraoperative findings showed bleeding from an intercostal artery during the surgery (**c**) and hemostasis was achieved using soft coagulation (**d**)

A 68-year-old man underwent right lower lobectomy for invasive mucinous adenocarcinoma (pT2aN1M0 Stage IIB) in June 2016 by an open thoracotomy approach. Postoperative follow-up computed tomography (CT) examinations revealed a pure-solid pulmonary nodule in the right upper lobe of his lung (Fig. 1a), which was found to be slightly enlarged on a follow-up CT evaluation (Fig. 1b). In July 2019, a partial pulmonary resection of the right upper lobe was performed. During the surgery, a severe adhesion between the lung and the chest wall was noted, and dissection of the adhesion caused bleeding from the 6th intercostal artery (Fig. 1c). Hemostasis was achieved using soft coagulation (Fig. 1d). Dissection of the adhesion was performed only to the extent necessary to perform the partial resection. The postoperative course was uneventful, and the patient was discharged 5 days after surgery.

**Fig. 2:**
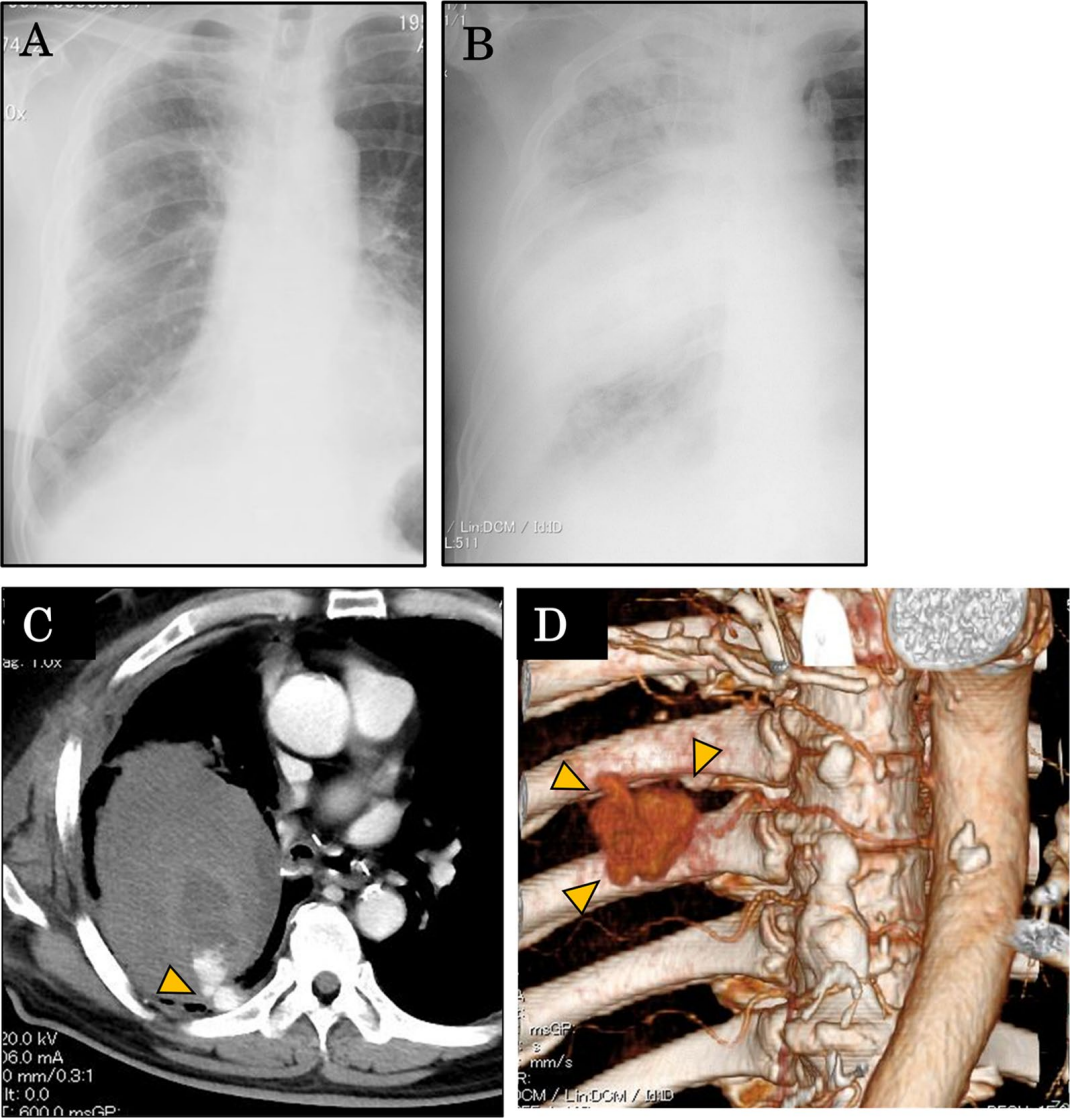
Radiological examinations performed on the day of late-onset bleeding (18 days after surgery). Chest X-rays before the bleeding (**a**) and after the emergency transportation (**b**) are shown. Contrast-enhanced CT examination revealed bleeding from the intercostal artery (arrowhead) and a large intrathoracic hematoma (**c**). The 3D reconstruction of the CT revealed a pseudoaneurysm (arrowhead) of the intercostal artery (**d**)

The first postoperative outpatient visit was 18 days after surgery, and blood test and chest X-ray (Fig. 2a) showed no abnormal findings. However, the patient later experienced hemoptysis at the toilet of a convenience store adjacent to the hospital. He was rushed to the hospital by medical staff who were assembled by a stat call. His vital scores were unstable; blood pressure (BP) was 117/98 mmHg, heart rate (HR) was 125 bpm, respiratory rate (RR) was 30 beats/min, and SpO_2_ was 70% (room air). A second chest X-ray on the same day showed decreased permeability in the right middle lung field (Fig. 2b). Contrast-enhanced CT scan of the chest showed a hematoma with a maximum diameter of 11 cm in the right thoracic cavity, a small amount of free air, and leakage of contrast medium into the hematoma from 6th intercostal artery (Fig. 2c). A 3D-reconstruction of the chest CT revealed a pseudoaneurysm of the intercostal artery (Fig. 2d).

**Fig. 3:**
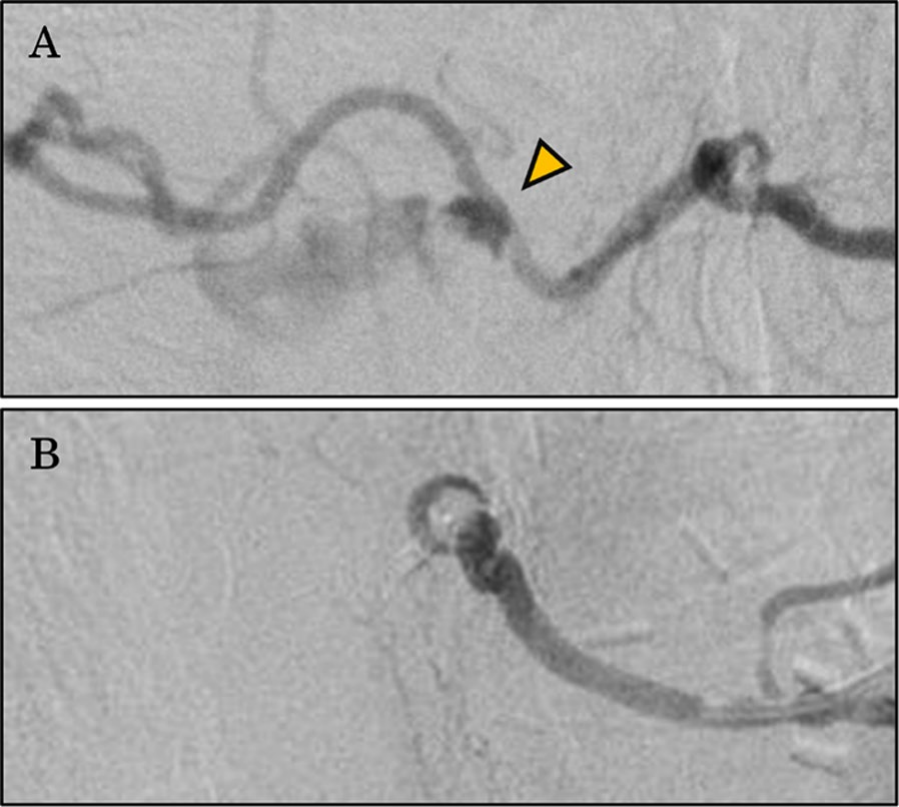
Successful hemostasis by angiographical embolization. Angiography successfully identified the bleeding point (arrowhead) of the intercostal artery (**a**). Embolization using *n*-butyl-2-cyanoacrylate plus lipiodol in a 1:3 mixture was performed, and hemostasis was obtained (**b**)

The patient was immediately admitted to the Radiology Department for emergency angiography. Because his systolic BP was 58 mmHg on admittance, a rapid transfusion of four units of red cell concentrate (RCC) was administered during the preparation for angiography. The angiography through the right femoral artery revealed extravasation from the 6th intercostal artery (Fig. 3a). Therefore, the 6th intercostal artery was embolized using an embolization agent (NBCA:lipiodol = 1:3) to stop the bleeding.

After the emergency angiography, the patient’s general condition recovered. However, a CT examination performed 2 days later found free air within the hematoma. Because pulmonary fistula was suspected, a chest drain tube (24Fr.) was inserted into the intrathoracic cavity. The air leak disappeared on day 17 after angiography and the patient was discharged on day 22.

## Discussion

Here we report a case of late-onset postoperative hemorrhage (postoperative 18th day) after partial resection of the lung. Hemoptysis, the symptom of the hemorrhage, occurred at a convenience store adjacent to the hospital, and medical staffs in our hospital rushed to the store in response to a stat call. It may be questioned whether the stat call was adequate at an out-of-hospital facility, we speculate that the quick transport of the patient might have saved his life as he was in a pre-shock state when he arrived at the angiography room. This may suggest that a rupture of a pseudoaneurysm of the intercostal artery can be a potentially fatal situation.

The primary symptom in this case was hemoptysis. In general, the differential diagnosis of hemoptysis after pulmonary resection includes bleeding from the staple line, pulmonary artery–bronchial fistula [7], and aspergillosis in the remaining cavity in the lung [8]. Because the patient received partial resection and 3 years had passed since he received right lower lobectomy, a pulmonary artery–bronchial fistula was unlikely. Therefore, a contrast-enhanced CT scan was immediately performed to investigate the bleeding point. We concluded that hemorrhage from the intercostal artery caused the hematoma, and the increased pressure within the hematoma caused a break of the adhesion between the lung and the chest wall, resulting in lung injury (pulmonary fistula). The patient may have inhaled the blood of the hematoma into the bronchus through the pulmonary fistula, leading to hemoptysis.

Postoperative intercostal artery pseudoaneurysm is a very rare complication. To the best of our knowledge, only two case reports have been reported thus far [9, 10]. Kawai et al. reported a case who experienced pseudoaneurysm of the 7th intercostal artery after video-assisted right lower lobectomy. The authors suspected that the pseudoaneurysm occurred at the site of a surgical port (with the possibility of injury of the intercostal artery during surgery). Intrathoracic hemorrhage was observed at postoperative days 4 and 18, and surgery was performed to control the hemorrhage on the 18th postoperative day [11]. The second case experienced a pseudoaneurysm caused by a biopsy of the parietal pleura (it is also possible that the intercostal artery was injured). In our case, we found that the intercostal artery was injured (Fig. 1C) and we used soft coagulation to stop the bleeding (Fig. 1D).

In the use of the coagulation mode of conventional electrocautery, hemostasis is achieved by burning the surface of the tissue. This leads to the formation of carbonized crusts; if the carbonized crusts detach, for example from mechanical stress, this can lead to postoperative bleeding. On the other hand, in soft coagulation, dehydrated dry tissues and denatured proteins are the main mechanisms that contribute to hemostasis; therefore, the risk of postoperative bleeding is lower than the coagulation mode of conventional electrocautery [12]. The soft coagulation used during the surgery (AMCO, Tokyo, Japan) is the one widely used in general, and was used with the standard soft coagulation mode output setting of Valleylab™ FT10 (Medtronic plc, Dublin, Ireland). However, while soft coagulation was used in our patient, intraoperative video recorded some degree of tissue carbonization likely due to the electrode being held in place a little longer than usual (Fig. 1D). We consider this may be one of the reasons for the vulnerability of the artery wall to form a pseudoaneurysm.

## Conclusions

Soft coagulation is widely used to control minor bleeding from small vessels during pulmonary resection. Soft coagulation is a useful because it provides rapid hemostasis without carbonizing the tissue. However, our experience in treating the current case indicates that additional hemostasis, such as the ligation at the proximal site of the hemorrhage point, would be considered for small arteries after hemostasis is achieved by soft coagulation, especially when some degree of tissue carbonization is suspected.

## Data Availability

All data are available from the corresponding author upon reasonable request.

## Abbreviations

CT: Computed tomography
BP: Blood pressure
HR: Heart rate
RR: Respiratory rate
RCC: Red cell concentrate
NBCA: *N*-Butyl-2-cyanoacrylate
